# Optical Sensor System for 3D Jones Matrix Reconstruction of Optical Anisotropy Maps of Self-Assembled Polycrystalline Soft Matter Films

**DOI:** 10.3390/s24051589

**Published:** 2024-02-29

**Authors:** Waldemar Wójcik, Zhengbing Hu, Yuriy Ushenko, Andrzej Smolarz, Iryna Soltys, Oleksander Dubolazov, Oleksander Ushenko, Olexandra Litvinenko, Ivan Mikirin, Ivan Gordey, Oleksandr Pavlyukovich, Sergii Pavlov, Natalia Pavlyukovich, Saltanat Amirgaliyeva, Aliya Kalizhanova, Zhalau Aitkulov

**Affiliations:** 1 Department of Electronics and Information Technology, Lublin University of Technology, 20-618 Lublin, Poland; waldemar.wojcik@pollub.pl; 2 School of Computer Science, Hubei University of Technology, Wuhan 430068, China; hzb@mail.ccnu.edu.cn; 3 Computer Science Department, Yurii Fedkovich Chernivtsi National University, 58012 Chernivtsi, Ukraine; y.ushenko@chnu.edu.ua (Y.U.); i.soltys@chnu.edu.ua (I.S.); o.ushenko@chnu.edu.ua (O.U.); mikirin.ivan@chnu.edu.ua (I.M.); hordei.ivan@chnu.edu.ua (I.G.); 4 Photoelectric Information Center, Research Institute of Zhejiang University, Taizhou 310058, China; 5 Department of Forensic Medicine and Medical Jurisprudence, Bukovinian State Medical University, 58000 Chernivtsi, Ukraine; o.litvinenko@bsmu.edu.ua (O.L.); o.pavlyuklovich@bsmu.edu.ua (O.P.); n.pavlyuklovich@bsmu.edu.ua (N.P.); 6 Laboratory of Biomedical Optics, Department of Biomedical Engineering and Optic-Electronic Systems, Faculty for Infocommunications, Radioelectronics and Nanosystems, Vinnytsia National Technical University, 21000 Vinnytsia, Ukraine; psv@vntu.edu.ua; 7 Academy of Logistics and Transport, Almaty 050012, Kazakhstan; saltanat_amirgal@mail.ru; 8 Institute of Information and Computational Technologies CS MES RK, Almaty 050010, Kazakhstan; kalizhanova_aliya@mail.ru (A.K.); jalau@mail.ru (Z.A.); 9 Department of IT Engineering, Institute of Automation and Information Technology, Almaty University of Power Engineering and Telecommunications, Almaty 050013, Kazakhstan; 10 Department of Information Technologies and Library Affairs, Institute of Physics, Mathematics and Computing, Kazakh National Women’s Teacher Training University, Almaty 050000, Kazakhstan

**Keywords:** Jones matrix, 3D reconstruction, blood plasma, optical anisotropy, diagnostics

## Abstract

Our work uses a polarization matrix formalism to analyze and algorithmically represent optical anisotropy by open dehydration of blood plasma films. Analytical relations for Jones matrix reconstruction of optical birefringence maps of protein crystal networks of dehydrated biofluid films are found. A technique for 3D step-by-step measurement of the distributions of the elements of the Jones matrix or Jones matrix images (JMI) of the optically birefringent structure of blood plasma films (BPF) has been created. Correlation between JMI maps and corresponding birefringence images of dehydrated BPF and saliva films (SF) obtained from donors and prostate cancer patients was determined. Within the framework of statistical analysis of layer-by-layer optical birefringence maps, the parameters most sensitive to pathological changes in the structure of dehydrated films were found to be the central statistical moments of the 1st to 4th orders. We physically substantiated and experimentally determined the sensitivity of the method of 3D polarization scanning technique of BPF and SF preparations in the diagnosis of endometriosis of uterine tissue.

## 1. Introduction

The relevance of diagnosis and treatment of prostate cancer is undeniable because of the high prevalence and hidden nature of the disease in the global male population [1,2,3]. The most important diagnostic task is the differentiation of prostate cancer stages, which determines the prognosis and treatment [4]. A variety of optical analysis techniques could be a promising avenue for addressing these pressing biomedical challenges [5,6,7,8,9,10]. The most sensitive to polarization manifestations of changes in the optical anisotropy of biological objects [11,12,13,14,15] are the multiparameter techniques of Mueller matrix [5,16,17], polarimetry [10,18,19,20,21,22,23], matrix decomposition [24,25] and coordinate mapping [26,27,28,29] using various analytical approximations [6,7,8,9], computer proceedings [30,31,32,33,34] and optically devises [35,36,37,38,39,40].

The most accurate (gold standard) and mandatory stage of diagnosis is histological and immunohistochemical examination of tumor biopsy, which allows us to make a final conclusion, determine the histological picture of the pathological condition with an accuracy that is close to the maximum (~100%) [41]. At the same time, these studies are not rapid, require the use of expensive reagents, are also largely subjective, and do not provide the possibility of quantitative analysis of the parameters of microscopic images of internal organ preparations.

Currently, instrumental high-precision (~95%) polarization interference methods for differential diagnosis of histological sections of prostate tumor biopsy have been developed, which are devoid of the above disadvantages of traditional histological examination [42].

At the same time, obtaining a biopsy is a traumatic and sometimes dangerous operation for patients’ health. Therefore, it is relevant to develop new rapid instrumental biophysical methods for differential diagnosis of prostate tumors using a minimally invasive procedure, an optical study of the polycrystalline structure of dehydrated films of biological fluids of human organs.

Polarimetric studies of the polarization manifestations of BF anisotropy make it possible to detect pathological changes in the quaternary and tertiary polycrystalline structure of basic proteins (albumin and globulin) in blood plasma and other biological fluids, which are inaccessible in the most common biochemical methods. In a series of works [43,44,45,46,47,48,49,50,51], the sensitivity of polarimetric methods to inflammatory, pathological and necrotic changes in the polycrystalline structure of films of blood plasma, urine, synovial fluid, and cerebrospinal fluid was demonstrated.

Despite such promising results in diagnosing the structure of biofluid films, there are still a number of unresolved problems. Among them are the following:

1. There is no unified (unified) analytical description of polarization manifestations of optical properties of complex phase and amplitude anisotropic structure of networks of biological crystals in the volume of dehydrated BF films.
2. Algorithms for polarization reconstruction of histograms of polycrystalline networks of biological crystal maps of various optical anisotropy mechanisms, birefringence and dichroism, have not been developed.
3. Dehydrated BF films exhibit a complex spatially inhomogeneous volumetric structure of networks of biological crystals. On the other hand, polarization methods provide integrally averaged information over the whole volume of the biological layer in the form of 2D matrix element distributions. As a consequence, the sensitivity of existing methods of matrix polarimetry does not provide the capability to detect spatially localized changes of optical anisotropy of networks of biological crystals.

There is no unified medical-physical approach to the objective evaluation of diagnostic efficiency of matrix polarimetry methods.

Thus, a new and urgent issue of matrix polarimetry is the development of the Jones matrix model of analytical description of optical anisotropy of various types of dehydrated films of biological fluids followed by determination of algorithmic relationships between parameters of optical anisotropy of dehydrated BF films and values of partial matrix elements.

This polarimetric approach can be developed and generalized into a synthesis of polarization interference mapping and digital holographic reconstruction of object fields of complex amplitudes for layer-by-layer reconstruction of optical anisotropy maps of dehydrated BF films. Therefore, to improve the sensitivity and accuracy of polarimetric matrix diagnostics, it is urgent to create a new method for the layer-by-layer study of polycrystalline films of biological fluids by synthesizing polarization (Jones matrix) and interference methods [43,44,45,46,47,48,49,50].

In addition, the potential of the Jones matrix tomography technique can be expanded to other equally relevant areas, such as instrumental medical diagnostics COVID-19 [51,52,53]

Our work aims to create and validate a new experimental method of polarization interference multilayer Jones matrix scanning of the polycrystalline structure of blood plasma (BPF) and saliva (SF) for diagnosis and differentiation of prostate cancer stages.

## 2. Methods and Theory

According to traditional concepts [54,55], a polycrystalline film of any biological fluid can be represented as a sequence of partial layers with six different types of phase (optical birefringence–LB_(0,90);(45,135)_, CB_⊗,⊕_) and amplitude (LD_(0,90);(45,135)_, CD_⊗,⊕_) anisotropy. These LD_0,90_, LD_45,135_ and LB_0,90_, LB_45,135_–“linear dichroism–birefringence”; CD_⊗;⊕_ and CB_⊗;⊕_–“circular dichroism–birefringence” of biological layer optically anisotropic component for linearly (0° ÷ 90° and 45° ÷ 135°) and circularly right-(⊗) and left-(⊕) polarised others.

(1) $$LB_{0;90}=\frac{2\pi }{\lambda }\left(n_{0}-n_{90}\right)h;$$

(2) $$LB_{45;135}=\frac{2\pi }{\lambda }\left(n_{45}-n_{135}\right)h;$$

(3) $$CB_{⊗;⊕}=\frac{2\pi }{\lambda }\left(n_{⊗}-n_{⊕}\right)h;$$

(4) $$LD_{0;90}=\frac{2\pi }{\lambda }\left(\chi_{0}-\chi_{90}\right)h;$$

(5) $$LD_{45;135}=\frac{2\pi }{\lambda }\left(\chi_{45}-\chi_{135}\right)h;$$

(6) $$CD_{⊗;⊕}=\frac{2\pi }{\lambda }\left(\chi_{⊗}-\chi_{⊕}\right)h;$$

where $\left(\begin{array}{cccc} n_{0}, & n_{90}, & n_{45}, & n_{135} \end{array}\right)$ and $\left(\begin{array}{cccc} \chi_{0}, & \chi_{90}, & \chi_{45}, & \chi_{135} \end{array}\right)$ are refractive indices–absorption for orthogonal amplitude components 0°–90° and 45°–135°; λ—laser wavelength; h—geometrical layer thickness.

Given the insignificant absorption of dehydrated BPF and SF in our work, we limit ourselves to taking into account the effects of birefringence.

We describe the polarization manifestations of each optical anisotropy mechanism by the Jones partial matrix operators of the formula below [48]:

(7) $$J\left(LB_{0;90}\right)=\left\|\begin{array}{cc} \exp\left(-0.5iLB_{0;90}\right) & 0\\ 0 & \exp\left(0.5iLB_{0;90}\right) \end{array}\right\|;$$

(8) $$J\left(LB_{45;135}\right)=\left\|\begin{array}{cc} \cos0.5LB_{45;135} & -i\sin0.5LB_{45;135}\\ -i\sin0.5LB_{45;135} & \cos0.5LB_{45;135} \end{array}\right\|;$$

(9) $$J\left(CB_{⊗;⊕}\right)=\left\|\begin{array}{cc} \cos CB_{⊗;⊕} & \sin CB_{⊗;⊕}\\ -\sin CB_{⊗;⊕} & \cos CB_{⊗;⊕} \end{array}\right\|;$$

An analytical form of the generalized Jones matrix of a polycrystalline medium with complex anisotropy was found in [48]

(10) $$\left\{J\right\}=\left\|\begin{array}{cc} j_{11} & j_{12}\\ j_{21} & j_{22} \end{array}\right\|=\left\|\begin{array}{cc} \cos0.5\left|V\right|-i\frac{L}{\left|V\right|}\sin0.5\left|V\right| & \frac{\left(C\;-\;iL^{\prime }\right)}{\left|V\right|}\sin0.5\left|V\right|\\ -\frac{\left(C\;+\;iL^{\prime }\right)}{\left|V\right|}\sin0.5\left|V\right| & \cos0.5\left|V\right|+i\frac{L}{\left|V\right|}\sin0.5\left|V\right| \end{array}\right\|$$

Here V—generalized anisotropy vector

(11) $$V=V\left(L_{0;90},L_{45;135};-C_{⊗;⊕}\right),$$

where

(12) $$\left\{\begin{array}{c} L_{0;90}=LB_{0;90};\\ L_{45;135}=LB_{45;135};\\ C_{⊗;⊕}=CB_{⊗;⊕}. \end{array}\right..$$

From (11) and (12) we obtain an explicit form of the vector modulus V

(13) $$\left|V\right|={\left(\left|L_{0;90}\right|+\left|L_{45;135}\right|+\left|C_{⊗;⊕}\right|\right)}^{0.5}\;={\left(\left(L{B^{2}}_{0;90}\right)+(L{B^{2}}_{45;135})+\left(C{B^{2}}_{⊗;⊕}\right)\right)}^{0.5},$$

On the basis of (7)–(13), we obtain theoretical interrelations between the parameters of linear and circular birefringence dichroism and elements of the Jones matrix $\left\{J\right\}$ of an optically anisotropic layer

(14) V=2arccos⁡0.5Re⁡j11+Re⁡j22;

(15) LB0;90=V2sin⁡0.5VIm⁡j22−Im⁡j11;

(16) LB45,135=V2sin⁡0.5VIm⁡j12+Im⁡j21;

(17) CB⊗;⊕=V2sin⁡0.5VIm⁡j12−Im⁡j21

The algorithm for calculating the elements of the Jones matrix $\left\{J\right\}$ (Expression (1)) includes the irradiation of a polycrystalline film sample by linearly polarised beams with azimuths 0° (Jones vector $\left(\begin{array}{c} 1\\ 0 \end{array}\right)$) and 90° (Jones vector $\left(\begin{array}{c} 0\\ 1 \end{array}\right)$)

(18) $$U^{0}=\left(\begin{array}{c} U_{x}^{0}\\ U_{y}^{o} \end{array}\right)=\left\|\begin{array}{cc} j_{11} & j_{12}\\ j_{21} & j_{22} \end{array}\right\|\left(\begin{array}{c} 1\\ 0 \end{array}\right)\Rightarrow \left(\begin{array}{c} j_{11}\\ j_{21} \end{array}\right)\Rightarrow \left\{\begin{array}{c} U_{x}^{0}=\left\|\begin{array}{cc} 1 & 0\\ 0 & 0 \end{array}\right\|\left(\begin{array}{c} j_{11}\\ j_{12} \end{array}\right)\Rightarrow j_{11};\\ U_{y}^{0}=\left\|\begin{array}{cc} 0 & 0\\ 0 & 1 \end{array}\right\|\left(\begin{array}{c} j_{11}\\ j_{12} \end{array}\right)\Rightarrow j_{21}; \end{array}\right.$$

(19) $$U^{90}=\left(\begin{array}{c} U_{x}^{90}\\ U_{y}^{90} \end{array}\right)=\left\|\begin{array}{cc} j_{11} & j_{12}\\ j_{21} & j_{22} \end{array}\right\|\left(\begin{array}{c} 0\\ 1 \end{array}\right)\Rightarrow \left(\begin{array}{c} j_{12}\\ j_{22} \end{array}\right)\Rightarrow \left\{\begin{array}{c} U_{x}^{90}=\left\|\begin{array}{cc} 1 & 0\\ 0 & 0 \end{array}\right\|\left(\begin{array}{c} j_{12}\\ j_{22} \end{array}\right)\Rightarrow j_{21};\\ U_{y}^{90}=\left\|\begin{array}{cc} 0 & 0\\ 0 & 1 \end{array}\right\|\left(\begin{array}{c} j_{12}\\ j_{22} \end{array}\right)\Rightarrow j_{22}. \end{array}\right.$$

As a result, we obtain the following expressions for the Jones matrix tomography of the polycrystalline structure of a biological fluid film

(20) $$\left|V\right|=2\arccos\left[0.5\left(U_{x}^{0}\exp\left(i\varphi_{x}^{0}\right)+U_{y}^{90}\exp\left(i\varphi_{y}^{90}\right)\right)\right];$$

(21) $$LB_{0;90}=\frac{\left|V\right|}{2\sin0.5\left|V\right|}\left(U_{y}^{90}\sin\varphi_{y}^{90}-U_{x}^{0}\sin\varphi_{x}^{0}\right);$$

(22) $$LB_{45,135}=\frac{\left|V\right|}{2\sin0.5\left|V\right|}\left(U_{y}^{0}\sin\varphi_{y}^{0}-U_{x}^{90}\sin\varphi_{x}^{90}\right);$$

(23) $$CB_{⊗;⊕}=\frac{\left|V\right|}{2\sin0.5\left|V\right|}\left(U_{y}^{0}\sin\varphi_{y}^{0}-U_{x}^{90}\sin\varphi_{x}^{90}\right);$$

In further experimental studies, we will use the generally accepted parameters for the different types of birefringence

(24) $$LB=\sqrt{LB_{0;90}^{2}+LB_{45;135}^{2}};$$

(25) $$CB\equiv CB_{⊗;⊕}.$$

## 3. Optical Scheme of 3D Jones Matrix Scanning of BPF and SF

**Figure 1:**
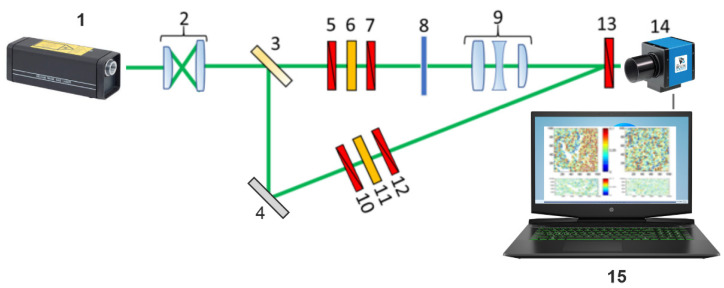
Optical scheme of optical sensor system for Jones matrix tomography of polycrystalline films of biological fluids. 1—HeNe laser (Ø = 2 × 10^3^ μm, λ = 0.6328 μm); 2—collimator; 3—light splitter (50%); 4—turning mirror; 5, 7, 10, 12, 13—linear polarizers; 6, 11—quarter wave plates; 8—sample of dehydrated film; 9—polarizing microobjective; 14—digital camera; 15—PC.

Figure 1 shows a diagram of the polarization interferometry of Jones layer-by-layer distributions of dehydrated films JMI [46,47,49].

## 4. The Method of 3D Jones Matrix Scanning of BPF and SF

1. Two states of linear polarisation are sequentially formed in the “irradiating” (Ir) and “reference” (Re) parallel laser beams–Ir(0°)−Re⁡(0°)≡p i Ir(90°)−Re⁡(90°)≡r.

2. For each of the polarization states (p and r), two partial interference patterns are recorded through the polarizer-analyzer 14 with the orientation of the transmission plane at angles $\begin{array}{cc} \Omega =0{}^{\circ}; & \Omega =90{}^{\circ} \end{array}$.

3. Analytical processing of microscopic interference images of dehydrated film samples was carried out using the digital Fourier transform $F\left(\upsilon ,\nu \right)$ [49]:

(26) $$F_{0;90}\left(\upsilon ,\nu \right)=\frac{1}{M\times N}\sum_{m=0}^{M-1}\sum_{n=0}^{N-1}I_{x,y}\left(\Omega =0{}^{\circ};90{}^{\circ}\right)\exp\left[-i2\pi \left(\frac{m\times \upsilon }{M}+\frac{n\times \nu }{N}\right)\right].$$

where

$$\left\{\begin{array}{c} I_{x}^{0}\left(\Omega =0{}^{\circ}\right)\left(m,n\right)=U_{x}^{0}\left(\Omega =0{}^{\circ}\right)\left(m,n\right)\left(U_{x}^{0}\right)*\left(\Omega =0{}^{\circ}\right)\left(m,n\right);\\ I_{x}^{90}\left(\Omega =90{}^{\circ}\right)\left(m,n\right)=U_{x}^{90}\left(\Omega =90{}^{\circ}\right)\left(m,n\right)\left(U_{x}^{90}\right)*\left(\Omega =90{}^{\circ}\right)\left(m,n\right)\\ I_{y}^{0}\left(\Omega =0{}^{\circ}\right)\left(m,n\right)=U_{y}^{0}\left(\Omega =0{}^{\circ}\right)\left(m,n\right)\left(U_{y}^{0}\right)*\left(\Omega =0{}^{\circ}\right)\left(m,n\right)\\ I_{y}^{90}\left(\Omega =90{}^{\circ}\right)\left(m,n\right)=U_{y}^{90}\left(\Omega =90{}^{\circ}\right)\left(m,n\right)\left(U_{y}^{90}\right)*\left(\Omega =90{}^{\circ}\right)\left(m,n\right) \end{array}\right.$$

$U_{x,y}^{0,90}$, orthogonal components of complex amplitude for different orientations, $\begin{array}{cc} \Omega =0{}^{\circ}; & \Omega =90{}^{\circ} \end{array}$; * denotes the complex conjugation operation; $\left(\upsilon ,\nu \right)$ are the spatial frequencies and $\left(m=1120,n=960\right)$ are the number of pixels of the CCD camera

4. The results of the digital Fourier transform (Relations (25), (26)) are used to obtain distributions of complex amplitudes according to the following algorithms:

(27) $$\left\{\begin{array}{c} \Omega_{0^{0}}\to \left|U_{x}^{0}\left(\Omega =0{}^{\circ}\right)\right|;\\ \Omega_{90^{0}}\to \left|U_{x}^{90}\left(\Omega =90{}^{\circ}\right)\right|\exp\left(i\left(\varphi_{x}^{90}-\varphi_{x}^{0}\right)\right); \end{array}\right.$$

(28) $$\left\{\begin{array}{c} \Omega_{0^{0}}\to \left|U_{y}^{0}\left(\Omega =0{}^{\circ}\right)\right|;\\ \Omega_{90^{0}}\to \left|U_{y}^{90}\left(\Omega =90{}^{\circ}\right)\right|\exp\left(i\left(\varphi_{y}^{90}-\varphi_{y}^{0}\right)\right) \end{array}\right.$$

5. By means of stepwise (Δθ) phase ($\theta_{k}$) scanning of the reconstructed field of complex amplitudes (Relations (27), (28)) using Algorithms (20)–(25), we obtain coordinate distributions of the optical birefringence parameters $JT\left(\theta_{k},m\times n\right)$.

## 5. Statistical Analysis of Maps of Linear and Circular Birefringence of Polycrystalline Films of Biological Fluids

The resulting set of optical anisotropy maps $f\equiv \left\{\begin{array}{c} LB\left(\theta_{k},m,n\right);\\ CB\left(\theta ,m,n\right) \end{array}\right.$ was analyzed in a statistical approach using the following algorithms to calculate mean ($Z_{1}$), variance ($Z_{2}$), skewness ($Z_{3}$) and kurtosis ($Z_{4}$) [43,44,45,46,47,48,49,50,51]

(29) $$\begin{array}{c} Z_{1}=\frac{1}{K}\sum_{j=1}^{K}f_{j};\\ Z_{2}=\sqrt{\frac{1}{K}\sum_{j=1}^{K}{\left(f^{2}\right)}_{j};}\\ Z_{3}=\frac{1}{Z_{2}^{3}}\frac{1}{K}\sum_{j=1}^{K}{\left(f^{3}\right)}_{j};\\ Z_{4}=\frac{1}{Z_{2}^{4}}\frac{1}{K}\sum_{j=1}^{K}{\left(f^{4}\right)}_{j}, \end{array}$$

where K=M×N—CCD pixels quantity.

## 6. Diagnostic Method

The search for the most sensitive phase plane was carried out as follows.

1. At the first stage:

- Discrete phase scanning was carried out in “maximum” increments $\Delta \theta_{k}^{max}=0.25\;\mathrm{rad}$.
- Using Relations (20)–(25), birefringence maps were calculated $f\equiv \left\{\begin{array}{c} LB\left(\theta_{k},m,n\right);\\ CB\left(\theta ,m,n\right) \end{array}\right.$.
- Central statistical moments $Z_{i=1;2;3;4}$ were calculated for the optical anisotropy distributions $f\equiv \left\{\begin{array}{c} LB\left(\theta_{k},m,n\right);\\ CB\left(\theta ,m,n\right) \end{array}\right.$.
- The differences between the obtained values of the statistical parameters were assessed ${\left(\Delta Z_{i}\right)}_{k}=Z_{i}\left(\theta_{j+1}^{max}\right)-Z_{i}\left(\theta_{j}^{max}\right)$.
- The phase interval $\Delta \theta^{*}=\left(\theta_{j+1}^{max}÷\theta_{j}^{max}\right)$ was determined, within which the monotonic increase in the value ends $\Delta Z_{i}={(Z}_{i}\left(\theta_{j+1}^{max}\right)-Z_{i}\left(\theta_{j}^{max}\right))\leq 0$.
- The macrointerval phase found $\Delta \theta^{*}$ was analyzed again in finer increments $\Delta \theta_{q}^{min}=0.05\;\mathrm{rad}$ − $\Delta Z_{i}=Z_{i}\left(\theta_{j+1}^{min}\right)-Z_{i}\left(\theta_{j}^{min}\right)$.
- The new (most sensitive) phase section $\theta^{*}$ was determined, in which $\Delta Z_{i}\left(\theta^{*}\right)=max$.

2. In this plane $\theta^{*}$, the mean $\Delta {\bar{Z}}_{i=1;2;3;4}^{*}$ and standard deviations $\sigma \left(\Delta Z_{i}^{*}\right)$ are determined

Therefore, prostate tumor diagnosis, for each of the statistical moments $Z_{i=1;2;3;4}$ the sensitivity

(30) $$(Se_{12}\left(f\right)=\frac{a_{12}}{a_{12}+b_{12}}100\%;\;Se_{34}\left(f\right)=\frac{a_{34}}{a_{34}+b_{34}}100),$$

specificity

(31) $$(Sp_{12}\left(f\right)=\frac{c_{12}}{c_{12}+d_{12}}100\%;\;Sp_{34}\left(f\right)=\frac{c_{34}}{c_{34}+d_{34}}100\%)$$

and balanced accuracy

(32) $$(Ac_{12}\left(f\right)=0.5\left(Se_{12}+Sp_{12}\right);\;Ac_{34}\left(f\right)=0.5\left(Se_{34}+Sp_{34}\right))$$

were calculated [56,57,58,59].

Here, $a_{12}\left(a_{34}\right)$ and $b_{12}\left(b_{34}\right)$ are the number of correct and incorrect diagnoses within investigation group 2 (group 4); and $c_{12}\left(c_{34}\right)$ and $d_{12}\left(d_{34}\right)$ are the same within control group 1 (group 3).

## 7. Biological Samples

Blood and saliva samples were taken for the purpose of determination of the prostate tumor [60,61,62,63].

Experimental blood plasma samples were obtained by centrifugation. Liquid drops with a volume of 10 μL were applied onto optically homogeneous glass heated to the temperature of the human body (36.6 °C). The process of complete drying (dehydration) lasted 45–50 min.

Experimental saliva samples were obtained by taking 2–3 mL of liquid into a test tube and settling it until the mucin proteins precipitated. Then, by centrifugation, the liquid and sedimentary components were separated. A drop of liquid with a volume of 10 μL was applied to optically homogeneous glass heated to the temperature of the human body (36.6 °C). The process of complete drying (dehydration) lasted 40–45 min.

The main biochemical components of blood plasma are water (90%), proteins (7%), organic nonprotein compounds (2%), salts (1%). Protein composition—60% albumin, 40% globulin.

It is known [62] that in the process of blood plasma dehydration, a dry (polycrystalline) drop is formed, which contains three zones:

- “outer”—an annular ridge, which almost entirely consists of linearly birefringent needlelike albumin crystals (h~12 μm−15 μm);
- “transitional”—circularly birefringent structure of globulin crystals with a small content of albumin crystals and optically isotropic crystals of NaCl salt (h~10 μm−12 μm);
- “Central”—solid crystals of NaCl salt (h~7 μm−10 μm).

The composition of saliva and blood plasma is correlated with each other. Saliva contains the same protein fractions as blood plasma—albumin and globulins. At the same time, among them, there are significantly fewer albumins and four times more globulins.

When a saliva drop is dehydrated, the crystallization results differ significantly from the polycrystalline structure of a blood plasma drop. This is due to the lower concentration of albumin and globulin proteins in saliva. Therefore, the marginal protein roll is not formed.

A dry drop of saliva has the form of a film quasi-uniform in thickness (h~10 μm÷15 μm), where optically isotropic dendritic networks of cubic crystals of NaCl salt are localized.

Such a film contains, in the form of domains, large (linearly birefringent needlelike albumin crystals) and small (circularly birefringent globulin crystals) optically anisotropic aggregates with a range of sizes h=10 μm÷500 μm.

Four representative groups of blood plasma and saliva polycrystalline films were formed:

- Group 1 consisted of n=36 samples of BPF from healthy donors;
- Group 2 consisted of n=36 samples of BPF films of patients with moderately differentiated adenocarcinoma (3 + 4; 4 + 3 on Gleason’s Pattern scale-ISUP grade 2–3);
- Group 3 consisted of n=36 SF samples from healthy donors.
- Group 4 consisted of n=36 SF samples from patients with moderately differentiated adenocarcinoma (3 + 4; 4 + 3 on Gleason’s Pattern scale-ISUP grade 2–3).

## 8. Results

At the first stage, layer-by-layer maps $f\equiv \left\{\begin{array}{c} LB\left(\theta_{k},m,n\right);\\ CB\left(\theta ,m,n\right) \end{array}\right.$ of BPF and SF of healthy donors were studied. The results of 3D Jones matrix layer-by-layer tomography for phase planes $\begin{array}{ccc} \theta_{k}=0.1\;\mathrm{rad}; & 0.25\;\mathrm{rad}; & 0.5\;\mathrm{rad} \end{array}$ are presented in the framework of statistical analysis of changes in coordinate distributions of the magnitude of optical birefringence parameters of supramolecular networks of biological crystals.

Such processes are quantitatively characterized by a set of mean values ${\bar{Z}}_{i}$ and standard deviations ±2σ (within group 1 and group 3) of the values of statistical moments $Z_{i}$ (Relation (29)) characterizing the maps of optical anisotropy $f\equiv \left\{\begin{array}{c} LB\left(\theta_{k},m,n\right);\\ CB\left(\theta ,m,n\right) \end{array}\right.$ of polycrystalline films of blood plasma and saliva (Table 1).

The data obtained revealed the individual means of all parameters $Z_{i}$, characterizing the statistical structure of BPF and SF maps $f\equiv \left\{\begin{array}{c} LB\left(\theta_{k},m,n\right);\\ CB\left(\theta ,m,n\right) \end{array}\right.$, in all phase planes, $\begin{array}{ccc} \theta_{k}=0.1\;\mathrm{rad}; & 0.25\;\mathrm{rad}; & 0.5\;\mathrm{rad} \end{array}$.

The largest phase ($\theta_{k}$) differences were found for the statistical moments of the 3rd and 4th orders, which characterize the skewness and kurtosis of the distributions of the optical anisotropy parameters $f\equiv \left\{\begin{array}{c} LB\left(\theta_{k},m,n\right);\\ CB\left(\theta ,m,n\right) \end{array}\right.$ of polycrystalline films of biological fluids:

- BPF-$\Delta Z_{3}\left(LB\right)=2.48;\Delta Z_{4}\left(LB\right)=2.02$ and $\Delta Z_{3}\left(CB\right)=2.86;\Delta Z_{4}\left(CB\right)=1.99$;
- SF-$\Delta Z_{3}\left(LB\right)=2.52;\Delta Z_{4}\left(LB\right)=2.03$ and $\Delta Z_{3}\left(CB\right)=2.84;\Delta Z_{4}\left(CB\right)=2.02$

## 9. Diagnosis of Prostate Cancer

**Figure 2:**
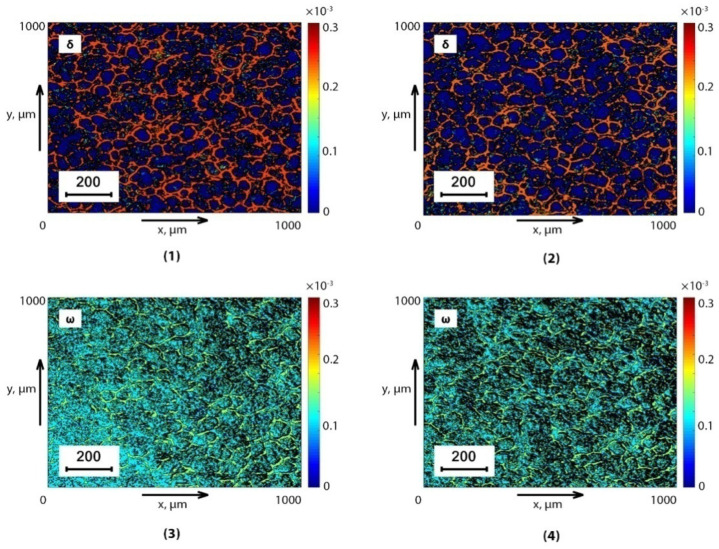
Topographic maps of linear $\theta_{blood}^{*}\left(LB\right)=0.35\mathrm{rad}$ (Fragments (1), (2)) and circular $\theta_{blood}^{*}\left(CB\right)=0.25\mathrm{rad}$ (Fragments (3), (4)) birefringence of healthy donors BPF (Fragments (1), (3)) and sick patients (Fragments (2), (4)).

**Figure 3:**
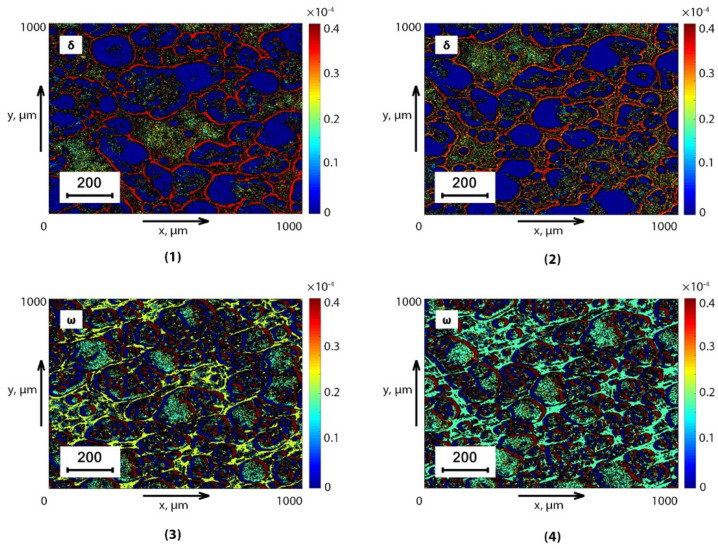
Topographic maps of linear $\theta_{blood}^{*}\left(LB\right)=0.35\mathrm{rad}$ (Fragments (1), (2)) and circular $\theta_{blood}^{*}\left(CB\right)=0.25\mathrm{rad}$ (Fragments (3), (4)) birefringence of SF of healthy donors (Fragments (1), (3)) and sick patients (Fragments (2), (4)).

The results of 3D Jones scanning of the BPF and SF from the control (group 1 and group 3) and experimental (group 2 and group 4) for the experimentally optimal phase planes ($\left\{\begin{array}{c} \theta_{blood}^{*}\left(\delta \right)=0.35\;rad;\\ \theta_{saliva}^{*}\left(\delta \right)=0.35\;rad \end{array}\right.$ and $\left\{\begin{array}{c} \theta_{blood}^{*}\left(\omega \right)=0.25\;rad;\\ \theta_{saliva}^{*}\left(\omega \right)=0.25\;rad; \end{array}\right.$) are shown in a series of fragments in Figure 2 and Figure 3.

On a series of fragments of Figure 2 and Figure 3 represent, reconstructed by Jones matrix tomography, coordinate distributions (optical anisotropy maps) of linear $LB\left(\theta^{*},m,n\right)$ (Fragments (1),(2)) and circular $CB\left(\theta^{*},m,n\right)$ (Fragments (3),(4)) birefringence of supramolecular networks of dehydrated plasma films (Figure 2) and saliva (Figure 3) of healthy donors (Fragments (1), (3)) and prostate cancer patients (Fragments (2), (4)).

Comparison of the obtained data revealed a correlation between the model analysis (Ratios (1)–(25)) and the experimental results (Figure 2 and Figure 3). Namely:

- The presence of mechanisms of linear $LB\left(\theta^{*},m,n\right)$ and circular $CB\left(\theta^{*},m,n\right)$ birefringence of supramolecular networks of dehydrated films of biological fluids with different morphological architectonics (Figure 2 and Figure 3).
- Individual topographic structure of maps of various birefringence mechanisms LB and CB, which are formed dendritic (Fragments (1), (2)) and spherulitic (Fragments (1), (2)) components of optically anisotropic supramolecular networks of biological crystals of dehydrated blood plasma films (Figure 2) and saliva (Figure 3).
- Insignificant differences (the magnitude of the average and the amplitude of fluctuations) between topographic maps of linear birefringence of $LB\left(\theta^{*},m,n\right)$ dendritic components of optically anisotropic architectonics of dehydrated plasma films (Figure 2) and saliva (Figure 3) of healthy donors (Fragments (1)) and prostate cancer patients (Fragments (2)).
- A significant increase in the circular birefringence of $CB\left(\theta^{*},m,n\right)$ spherulite components of the optically anisotropic architectonics of dehydrated plasma films (Figure 2) and saliva (Figure 3) of healthy donors (Fragments (3)) and prostate cancer patients (Fragments (4)).

Quantitative and objective scenarios of changes in various types of birefringence $LB\left(\theta^{*},m,n\right)$ and $CB\left(\theta^{*},m,n\right)$. The optically anisotropic architectonics of dehydrated films of biological fluids of both types are illustrated by statistical analysis data (ratios (29)), which are presented in Table 2.

The maximum intergroup differences were established:

- BPF-$\Delta Z_{3}\left(CB\right)=1.62;\Delta Z_{4}\left(CB\right)=1.63$;
- SF-$\Delta Z_{3}\left(CB\right)=1.69;\Delta Z_{4}\left(CB\right)=1.65$.

For these statistical parameters, Table 3 shows the values of the sensitivity Se, specificity Sp and balanced accuracy Ac of the method of layer-by-layer Jones matrix mapping with reproduction of maps of linear and circular birefringence of polycrystalline films of blood plasma and saliva.

The results shown in Table 3 indicate a different efficiency in diagnosing prostate cancer by Jones matrix layered reproduction of maps of linear $LB\left(\theta^{*},m,n\right)$ and circular $CB\left(\theta^{*},m,n\right)$ birefringence of BPF and SF.

It was found that the layer-by-layer mapping of the linear birefringence distributions $LB\left(\theta^{*},m,n\right)$ of the dendritic component of the optically anisotropic architectonics of dehydrated plasma films is quite sensitive and accurate (87.5%) to the pathological change in the structure of supramolecular networks of needlelike albumin crystals.

The accuracy of a similar diagnosis for the dendritic component of the supramolecular network of the dehydrated saliva film appears to be significantly lower (76.4%) due to a lower level of linear birefringence of needle-shaped albumin crystals and a high concentration of optically isotropic cubic NaCl crystals.

The most optimal and practically independent of the type of biological fluid was the statistical analysis of the coordinate distributions of the circular birefringence value $CB\left(\theta^{*},m,n\right)$ associated with the concentration of globulins. In this situation, an excellent level of balanced accuracy was achieved: $Ac\left(Z_{3;4}\right)=93.05\%÷95.8\%$.

## 10. Conclusions

For the first time, the Jones matrix analytical model for the optical anisotropy description of different types of dehydrated films of biological fluids, such as blood plasma and saliva, is proposed. New algorithmic relationships between phase anisotropy parameters (birefringence and optical activity) of dehydrated films and values of partial elements of the Jones matrix were found. Based on the synthesis of polarization interference mapping and digital holographic reconstruction of object fields of complex amplitudes, a new technique of phase Jones matrix scanning with the subsequent layer-by-layer reconstruction of optically mapped optically anisotropic structure of dehydrated biological liquid films was developed and tested experimentally for the first time. An original algorithm for determining the cross section in the volume of dehydrated films of biological fluids is proposed, which is most effective for differential diagnosis of pathological changes in optical birefringence. New objective criteria (markers) for high accuracy (93.05% to 95.8%) diagnosis and differentiation (91.7% to 94.4%) of prostate cancer stages have been defined in the statistical Jones matrix analysis of the reconstructed optical anisotropy maps of dehydrated blood plasma and saliva samples.

The present study is one of the first steps in the fundamental development and experimental testing of the diagnostic potential of the Jones express technique matrix tomography of polycrystalline architectonics of dehydrated human fluid films.

The next steps in the development of this method will be experimental studies planned on the scale of clinical institutions of the countries represented by the members of the author’s team for the purpose of expert evaluation and determination of the validity of Jones matrix tomography of pathological conditions of human organs.

## Figures and Tables

**Table 1:** Statistical parameters of optical anisotropy maps of BPF and SF of healthy donors.

f	$LB\left(\theta_{k},m,n\right)$		$CB\left(\theta_{k},m,n\right)$	
$Z_{i}$	Blood Plasma Group 1 *n* = 36	Saliva Group 3 *n* = 36	Blood Plasma Group 1 *n* = 36	Saliva Group 3 *n* = 36
$\theta_{k}$	θ=0.1 rad			
$Z_{1}\times 10^{-3}$	0.15 ± 0.012	0.021 ± 0.0011	0.09 ± 0.006	0.08 ± 0.003
$Z_{2}\times 10^{-3}$	0.12 ± 0.008	0.015 ± 0.0007	0.07 ± 0.008	0.06 ± 0.002
$Z_{3}$	1.54 ± 0.068	2.29 ± 0.11	2.12 ± 0.105	1.96 ± 0.088
$Z_{4}$	2.18 ± 0.098	3.11 ± 0.14	2.81 ± 0.13	2.73 ± 0.12
$\theta_{k}$	θ=0.25 rad			
$Z_{1}\times 10^{-3}$	0.24 ± 0.011	0.031 ± 0.0014	0.14 ± 0.007	0.12 ± 0.006
$Z_{2}\times 10^{-3}$	0.16 ± 0.008	0.017 ± 0.0007	0.11 ± 0.008	0.09 ± 0.004
$Z_{3}$	1.12 ± 0.068	1.62 ± 0.084	1.31 ± 0.061	1.22 ± 0.056
$Z_{4}$	1.51 ± 0.068	2.42 ± 0.12	2.13 ± 0.11	2.04 ± 0.105
$\theta_{k}$	θ=0.5 rad			
$Z_{1}\times 10^{-3}$	0.33 ± 0.014	0.039 ± 0.0015	0.16 ± 0.007	0.14 ± 0.006
$Z_{2}\times 10^{-3}$	0.21 ± 0.009	0.015 ± 0.0007	0.12 ± 0.005	0.09 ± 0.004
$Z_{3}$	0.62 ± 0.029	0.91 ± 0.044	0.74 ± 0.031	0.69 ± 0.028
$Z_{4}$	1.08 ± 0.045	1.53 ± 0.072	1.41 ± 0.064	1.35 ± 0.061

**Table 2:** Statistical parameters of optical anisotropy maps in sections $\theta^{*}\left(LB\right)=0.35\mathrm{rad}$ and $\theta^{*}\left(CB\right)=0.25\mathrm{rad}$ fields of complex amplitudes of BPF and SF.

$\theta_{k}$	$\theta^{*}\left(LB\right)=0.35\mathrm{rad}$		$\theta^{*}\left(CB\right)=0.25\mathrm{rad}$	
$Z_{i}$	**Blood Plasma Group 1** ***n* = 36**	**Blood Plasma Group 2** ***n* = 36**	**Blood Plasma Group 1** ***n* = 36**	**Blood Plasma Group 2** ***n* = 36**
$Z_{1}\times 10^{-3}$	0.24 ± 0.011	0.19 ± 0.009	0.14 ± 0.007	0.21 ± 0.011
$Z_{2}\times 10^{-3}$	0.16 ± 0.008	0.13 ± 0.007	0.11 ± 0.008	0.16 ± 0.009
$Z_{3}$	1.12 ± 0.068	1.45 ± 0.076	1.31 ± 0.061	0.81 ± 0.036
$Z_{4}$	1.51 ± 0.068	2.05 ± 0.108	2.13 ± 0.11	1.31 ± 0.09
$Z_{i}$	**Saliva** **Group 3** ***n* = 36**	**Saliva** **Group 4** ***n* = 36**	**Saliva** **Group 3** ***n* = 36**	**Saliva** **Group 4** ***n* = 36**
$Z_{1}\times 10^{-3}$	0.031 ± 0.0014	0.027 ± 0.0011	0.12 ± 0.006	0.17 ± 0.008
$Z_{2}\times 10^{-3}$	0.017 ± 0.0007	0.015 ± 0.0006	0.09 ± 0.004	0.14 ± 0.006
$Z_{3}$	1.62 ± 0.084	1.88 ± 0.094	1.22 ± 0.056	0.72 ± 0.035
$Z_{4}$	2.42 ± 0.12	2.84 ± 0.14	2.04 ± 0.105	1.24 ± 0.075

**Table 3:** Characteristics of the diagnostic power of the 3D matrix scanning method of BPF and SF.

w	$LB\left(\theta^{*}=0.35\mathrm{rad}\right)$		$CB\left(\theta^{*}=0.25\mathrm{rad}\right)$	
Groups	Group 1–Group 2	Group 3–Group 4	Group 1–Group 2	Group 3–Group 4
*Se* (%)	88.9	75	94.4	97.2
*Sp* (%)	86.1	77.8	91.7	94.4
*Ac* (%)	87.5	76.4	93.05	95.8

## Data Availability

The data presented in this study are available on request from the corresponding author. As the research involves human tissue, the availability of data is subject to the decision of the relevant ethics committee.
